# Calcite Dissolution by *Brevibacterium* sp. SOTI06: A Futuristic Approach for the Reclamation of Calcareous Sodic Soils

**DOI:** 10.3389/fpls.2016.01828

**Published:** 2016-12-08

**Authors:** S. M. Tamilselvi, Chitdeshwari Thiyagarajan, Sivakumar Uthandi

**Affiliations:** ^1^Biocatalysts Lab, Department of Agricultural Microbiology, Tamil Nadu Agricultural UniversityCoimbatore, India; ^2^Department of Soil Science and Agricultural Chemistry, Tamil Nadu Agricultural UniversityCoimbatore, India

**Keywords:** calcite dissolution, *Brevibacterium* sp., *in-vitro* analysis, calcareous soils, sodicity reclamation

## Abstract

Assessing the ability of soil microorganisms to dissolute poorly soluble native calcite to supply Ca^2+^ is a new area to be explored in reclaiming sodic soils by supplying adequate Ca^2+^ and reducing the recurrent sodicity. Hence, the present study aimed to isolate a calcite dissolving bacteria (CDB) from calcareous sodic soils and to understand the mechanism of calcite dissolution. Of the 33 CDB isolates recovered from the calcareous sodic soils of Tamil Nadu (Coimbatore, Ramnad, and Trichy), 11 isolates were screened for calcite dissolution based on titratable acidity. 16S rRNA gene sequence analysis of the three best isolates *viz*., SORI09, SOTI05, and SOTI06 revealed 99% similarity to *Bacillus aryabhattai*, 100% to *B. megaterium*, and 93% to *Brevibacterium* sp., respectively. Among them, *Brevibacterium* sp. SOTI06 released more Ca^2+^ (3.6 g.l^−1^) by dissolving 18.6% of the native calcite. The spectral data of FTIR also showed reduction in the intensity of calcite (55.36–41.27) by the isolate at a wave number of 1636 cm^−1^ which confirmed the dissolution. Besides producing organic acids (gluconic acid and acetic acid), *Brevibacterium* sp. SOTI06 also produced siderophore (91.6%) and extracellular polysaccharides (EPS, 13.3 μg. ml^−1^) which might have enhanced the calcite dissolution.

## Introduction

Soil degradation due to sodicity is the widest stress observed worldwide since the presence of high Na^+^ concentration increases the inter particulate distances by enhancing the repulsive forces and therefore causes dispersion and loss of porosity, which consequently results in undesirable soil structure and reduced water permeability in the soil profile. Many of these soils are highly deficient in plant nutrients due to high pH, exchangeable Na^+^, carbonates and bicarbonates, as a consequence crop production in these soils is also very poor (Murtaza et al., 2013; Tazeh et al., 2013). Hence, reclamation of these soils necessitates the removal of excess soluble Na^+^ from the soil to facilitate better crop growth.

Most of the sodic soils are calcareous in nature contains inherent or precipitated sources of Ca^2+^ in the form of calcite within the soil profile and such soils are widely spread in arid and semi arid regions. Calcite dissolution results in the release of Ca^2+^ ions to the soil solution (Qadir et al., 2007) which replace Na^+^ as detailed below (Qadir et al., 2005).

(1) $$\begin{array}{l} 2\text{N}{\text{a}}^{+}-\text{Clay }+\text{ C}{\text{a}}^{2+}\Leftrightarrow \text{C}{\text{a}}^{2+}-\text{Clay }+\;2\text{N}{\text{a}}^{+} \end{array}$$

Therefore, reclamation of calcareous sodic soils is possible when suitable amendments were identified and used at appropriate amounts. Generally amelioration of these soils has been achieved through the application of chemical amendments like gypsum (Abdel-Fattah, 2012; Cucci et al., 2012) as a direct source to supply sufficient Ca^2+^ for exchanging Na^+^. However, high cost and recurrent sodicity necessitates in finding out alternate sources and strategies. Phyto-remediation, a low cost technology involving different crops like kallar grass, sesbania, cotton, and halophytes like *Aster* sp., *Atriplex* sp., and *Plantago* sp. (Murtaza et al., 2009; Hasanuzzaman et al., 2014) helps to certain extent in lowering the sodicity but requires suitable plants, several growing seasons, and act only at limited depths (USEPA, 2000). Recently, microbial mediated calcite dissolution is gaining acceptance to reduce the sodicity.

However, most of the calcite dissolution mechanism has been studied without microorganisms (MacInnis and Brantley, 1992; Newton and Manning, 2002; Cucci et al., 2012) and only a very few reports have focused on the calcite dissolution by microorganisms (Lüttge and Conrad, 2004; Li et al., 2005; Jacobson and Wu, 2009; Subrahmanyam et al., 2012; Cacchio et al., 2014). Several mechanisms were reported for the extent of calcite dissolution such as acidification (Whitelaw et al., 1999) by producing organic acids (Goldstein, 1995; Fasim et al., 2002; Chen et al., 2006), inorganic acids (Hopkins and Whiting, 1916), chelating substances (Liermann et al., 2000; Yoshida et al., 2002), EPS (Yi et al., 2008), etc. Despite many reports on the mechanism of calcite dissolving microorganisms, it mainly centered around the production of organic acids like acetic acid, lactic acid, propionic acid, pyruvic acid, and succinic acid (Garcia-Pichel, 2006; Sulu-Gambari, 2011), enzymes like phosphatase (Ehrlich et al., 2008), EPS (Bissett et al., 2011) but none of them revealed the quantitative data on calcite dissolution. Hence, the present investigation aimed to isolate, identify an efficient CDB and measure their *in-vitro* calcite dissolution ability with an intention of using them for bio-remediating the calcareous sodic soils.

## Materials and methods

### Materials

Organic acids were from Sigma-Aldrich, India (Bengaluru) and other organic, inorganic analytical grade chemicals and agarose were from HI-Media Laboratories Pvt. Ltd. (Mumbai). Molecular biology chemicals were from New England Biolabs (Gurgaon, India) and Takara India (New Delhi).

### Media and cultivation conditions

Unless and otherwise stated all the culture conditions were performed in 100 ml of DB (Devenze-Bruni) medium in 250 ml Erlenmeyer flasks (with final OD_600 nm_ of 0.1) containing CaCO_3_ (5 g.l^−1^) and incubated at 30°C under shaking at 120 rpm for 24 h. The cell free culture supernatant obtained by centrifugation at 8000 g for 15 min was used for analysis of pH, TA, Ca^2+^, CaCO_3_, ${\text{CO}}_{3}^{2-}$, ${\text{HCO}}_{3}^{-}$, acid phosphatase, organic acid, EPS, biofilm, and siderophore.

### Isolation, screening, and identification of calcite dissolving bacteria

#### Soil sampling and enrichment

Calcareous sodic soil samples collected from three districts of Tamil Nadu, India *viz*., Coimbatore (Altitude of 411 m above mean sea level, 11.0°N latitude and 76.9°E longitude), Ramnad (Altitude of 2 m, 9.3°N latitude and 78.8°E longitude), and Trichy (Altitude of 85 m, 10.7°N latitude and 78.7°E longitude), showed the free CaCO_3_ concentration of 7.2, 7.6, and 7.8%, respectively and were stored at 4°C. In order to isolate CDB, 100 g of each soil was enriched with 1% CaCO_3_ individually and incubated for 2 weeks. Along with enriched soil samples, native, or initial soil samples were also used for the isolation of CDB.

#### Isolation and screening of CDB isolates

The CDB were isolated from both enriched and initial soil samples by serial dilution and plating technique using DB agar medium consisting of g.l^−1^ Glucose 5; Yeast extract 1; Peptone 1; K_2_HPO_4_ 0.4; MgSO_4_ 0.01; NaCl 5; (NH_4_)_2_SO_4_ 0.05; CaCO_3_ 5 and Agar 20 (Cacchio et al., 2004). The CD positive isolates picked based on clear zone formation around the colony were further confirmed by point inoculation onto the same medium. The solubilization index (SI) of the individual isolates was determined by measuring the ratio of the clear zone and colony size on DB agar plate by using the following formula:

$$\begin{array}{l} \text{Solubilization index}=\frac{\text{Clear zone }+\text{ Colony size}}{\text{Colonysize}}––––\left[\text{F}1\right] \end{array}$$

(Mihalacheet al., 2015)

Secondary screening of positive isolates was carried out by calculating titratable acidity (TA) from 24 h old cultures grown in DB liquid medium. One milliliter of the cell free culture supernatant was titrated against 10 mM NaOH in the presence of phenolphthalein indicator until the appearance of pink color (Whitelaw et al., 1999).

#### Identification of CDB isolates by 16S rRNA

Total genomic DNA of the selected isolates were extracted and purified using the method described by Clark (2013). CDB isolates were identified by amplification of 16S rRNA gene using 27F (5′ AGAGTTTGATCCTGGCTCAG 3′) and 1492R (5′ GGTTACCTTGTTACGACTT 3′) primers with the PCR conditions of initial denaturation at 95°C for 10 min followed by 35 cycles of denaturation at 94°C for 30 s, annealing at 55°C for 30 s and extension at 72°C for 1 min, followed by a final extension at 72°C for 15 min in a thermo cycler (BioRad, USA). Then, the PCR products were cloned into the pGEMT vector and transformed into chemically competent *E. coli* DH5α cells (Sambrook et al., 1989). Positive clones were selected based on blue-white screening from Amp-X-gal-IPTG plates and further confirmed by colony lysis PCR using M13 forward (5′ GTAAAACGACGGCCAGT 3′) and reverse primers (5′ AACAGCTATGACCATG 3′). The positive clones were sequenced [Bioserve Biotechnologies (I) Pvt. Ltd., Hyderabad, India]. 16S rRNA gene sequence obtained for each clone was aligned and compared with available sequences of bacterial lineage using Ez Taxon-e (http://eztaxon-e.ezbiocloud.net/). A phylogenetic tree was constructed using MEGA 6 program (Tamura et al., 2013) and their grouping sequence was based on confidence values obtained by bootstrap analysis of 1000 replicates.

### Surface attachment of *Brevibacterium* sp. SOTI06

#### Biofilm (planktonic) formation

One day old *Brevibacterium* sp. SOTI06 culture (0.1 ml) was taken into 96 well micro titre plate, covered and incubated at 30°C for 24 h. After incubation, the plates were washed thoroughly with sterile distilled water and air dried. One hundred and fifty microliters of 0.1% crystal violet was added to each well and incubated for 45 min. The excess stain was removed by sterile distilled water and air dried. Subsequently, 200 μl of 95% ethanol was added to each well and plates were incubated for 10–15 min. Contents of each well were mixed and 125 μl of the crystal violet/ethanol solution was transferred to a separate clear bottom well and optical density was measured at 600 nm using micro plate reader (Molecular Devices LLC, USA; Djordjevic et al., 2002).

#### EPS production

*Brevibacterium* sp. SOTI06 was cultured in DB liquid medium supplemented with CaCO_3_ (5 g.l^−1^) and incubated at 30°C for 24 h at 120 rpm. The culture was centrifuged at 4000 g for 15 min and the pellet was used for estimation of EPS by suspending the pellet with 5 ml distilled water and 5 ml 0.1 N KOH. The contents were boiled at 100°C for 10 min. After cooling, the suspension was neutralized with 1M HCl and 1 ml of suspension, was mixed with 5 ml Anthrone reagent and the intensity of color was measured at 620 nm in UV-VIS spectrophotometer (Systronics, India, DuBois et al., 1956).

### Calcite dissolution (CD) potential of *Brevibacterium* sp. SOTI06

#### Estimation of dissolution

In order to quantify CD ability of *Brevibacterium* sp. SOTI06 grown in DB liquid medium, cell free culture supernatant obtained at periodical intervals were subjected to the analysis of calcium (Jackson, 2005), calcium carbonate (Piper, 1944), carbonates, bicarbonates (Richards, 1954), phosphatase (Tabatabai and Bremner, 1969), protein concentration (Bradford, 1976), pH, and TA.

#### Quantification of organic acid production

Organic acid production was estimated from 24 h old culture by injecting 30 μl of 0.2 μm filtered cell free supernatant in HPLC with a UV detector set at 210 nm. The organic separation was carried out on Cosmosil packed column (Nacalai Tesque, Japan) with 10.8% Acetonitrile in 0.0035 M H_2_SO_4_ as mobile phase at a flow rate of 0.6 ml.min^−1^ (Chen et al., 2006). The data integration and analysis was done using Autochrom software. HPLC grade organic acids kit (No.47264 from Sigma Aldrich, USA) was used as standards.

#### Analysis of CD by ATR-FT-IR

FT-IR spectrum of CaCO_3_ in the spent medium by *Brevibacterium* sp. SOTI06 was recorded in JASCO FT-IR 6800 fitted with diamond enabled Attenuated Total Reflectance (ATR) sample holder and a DLaTgs detector and compared with CaCO_3_. The wavelength range was from 400 to 4000 cm^−1^. Spectral measurements were done in triplicates and 64 scans were recorded for all samples at a 4 cm^−1^ resolution.

#### Siderophore production

Siderophore production of *Brevibacterium* sp. SOTI06 was observed by point inoculation with fresh culture onto Chrome Azural S (CAS) agar plate and incubated for 48 h at 30°C (Schwyn and Neilands, 1987), which was further confirmed by broth assay. The assay was carried out by mixing the culture supernatant (0.5 ml) with 0.5 ml CAS reagent and the absorbance was measured at 630 nm against a reference consisting of un-inoculated liquid medium. Siderophore content was estimated using the formula:

$$\begin{array}{l} \text{Per cent siderophore units}=\left(\text{Ar}-\text{As}/\text{Ar}\right)\times 100––––\left[\text{F}2\right] \end{array}$$

(Payne, 1994)

Where, Ar is the absorbance of reference and As is the absorbance of sample.

### Statistical analysis

All the data were subjected to statistical analysis in Microsoft Excel (Windows 2007) add-in with XLSTAT version 2010.5.05 (XLSTAT, 2010).

## Results

### Isolation, screening, and identification of CDB isolates

**Figure 1:**
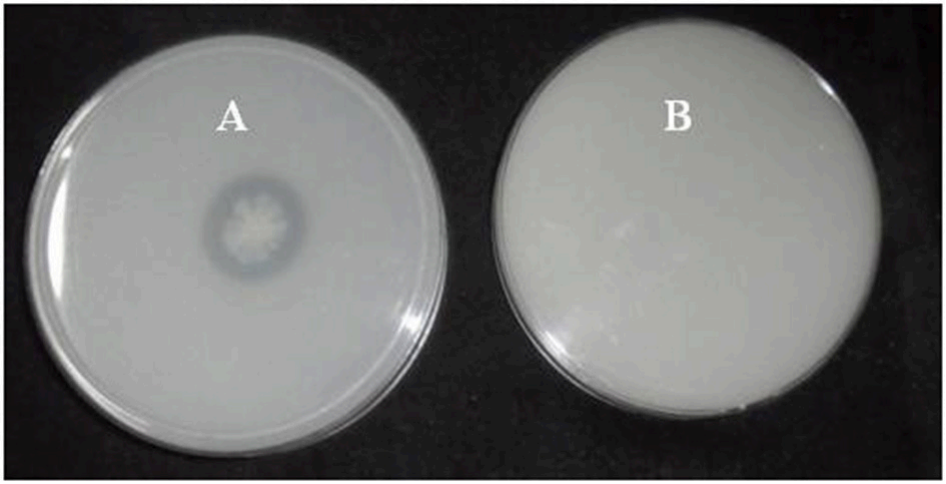
**Clear zone formation by ***Brevibacterium*** sp. SOTI06**. The bacterium forms a clear zone around the colony on DB medium in the presence of CaCO_3_ **(A)** indicating calcite dissolution was compared with control plate **(B)**.

**Figure 2:**
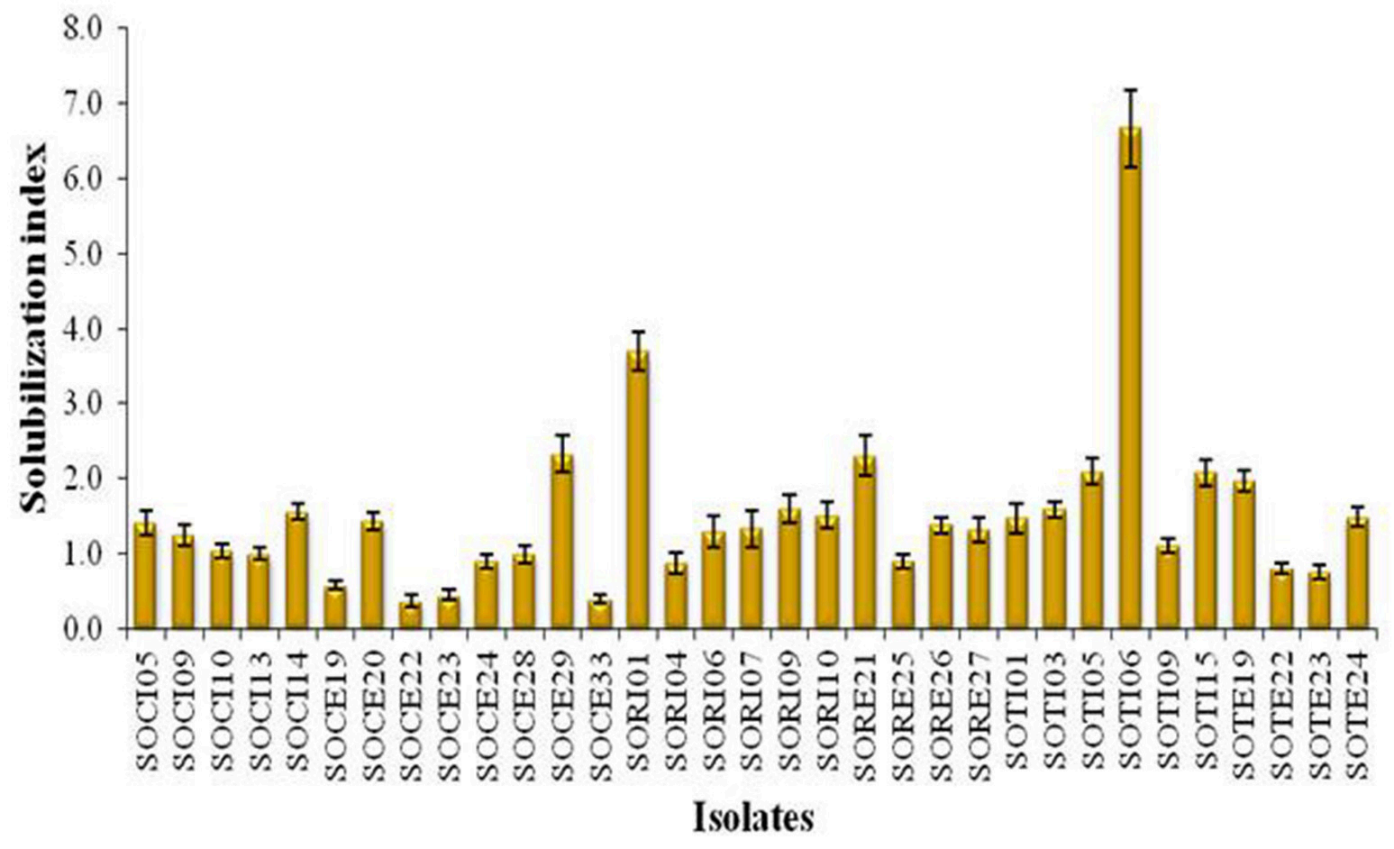
**Solubilization index of CDB isolates obtained from Tamil Nadu**. The SI was estimated for the isolates obtained from both initial and enriched soils of Coimbatore, Ramnad, and Trichy districts of Tamil Nadu. Means of three replicate values plotted and error bars indicate the standard error.

**Figure 3:**
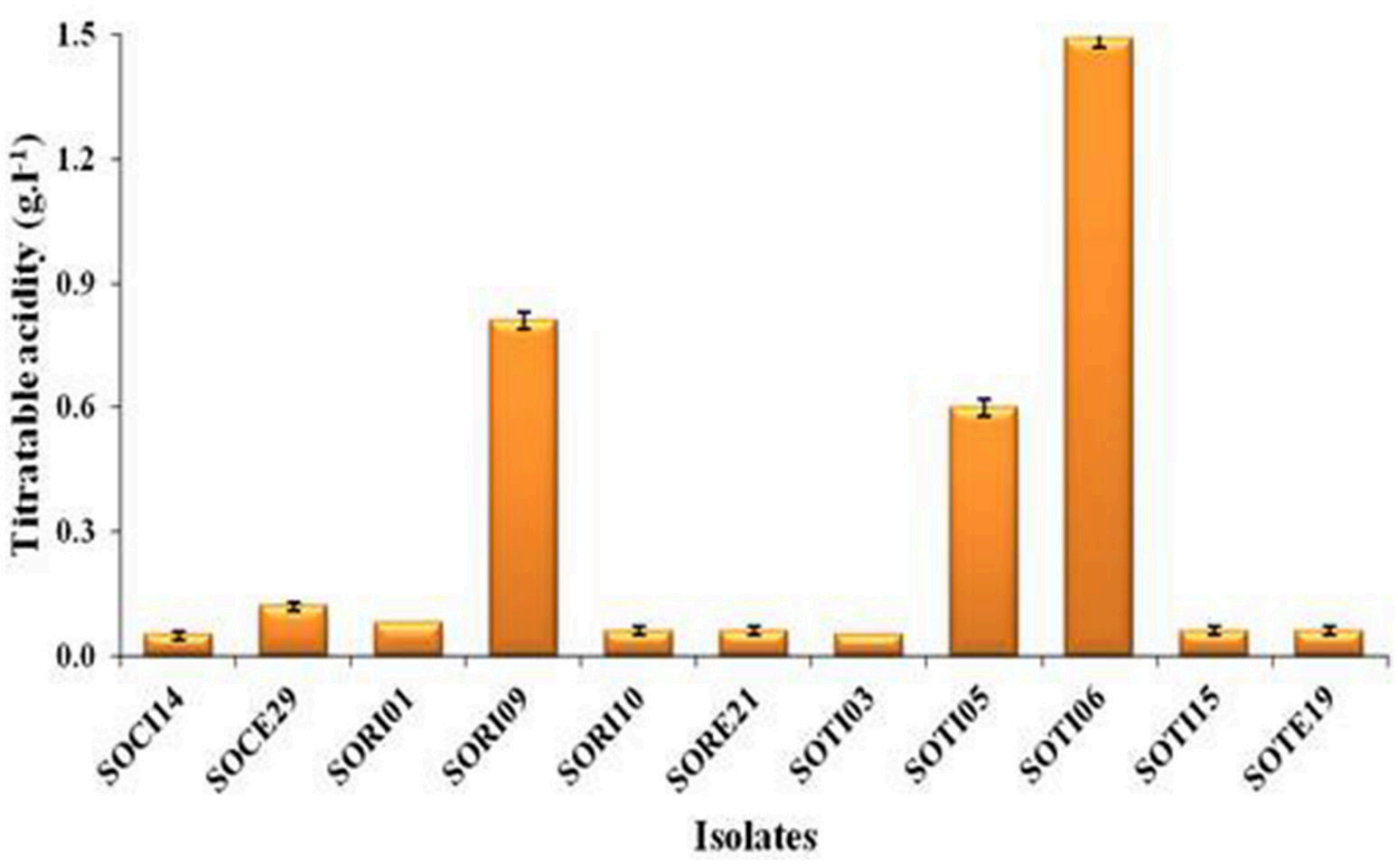
**Titratable acidity of selected CDB isolates**. The TA was determined from the culture supernatant of the isolates and the content was higher when these isolates produce acids. Means of three replicate values plotted and error bars indicate the standard error.

A total of 33 isolates (17 from native and 16 from enriched soils) showing clear zone (Figure 1) in DB medium was evaluated for calcite solubilization index (SI) which varied from 0.37 to 6.67. Among the three soils, SI values were higher with the isolates from Trichy soil than in Coimbatore and Ramnad soils. Higher SI values were observed in native isolates (0.88–6.67) compared to isolates from enriched soils (0.37–2.33). Among the isolates, SOTI06 showed maximum calcite SI (6.67) followed by SORI01 (3.70) and SOTI05 (2.10) which were from initial soils. On the other hand, maximum SI of 2.33 was observed in enriched isolate SOCE29. The least SI was recorded for the isolates SOCE22 and SOCE33 (Figure 2). Top 11 isolates having the highest SI were evaluated for TA production ability. Among them, eight isolates produced TA in the range of 0.05–0.12 g.l^−1^ and three isolates, SORI09, SOTI05, and SOTI06 produced maximum TA of 0.81, 0.60, and 1.41 g.l^−1^, respectively (Figure 3).

**Figure 4:**
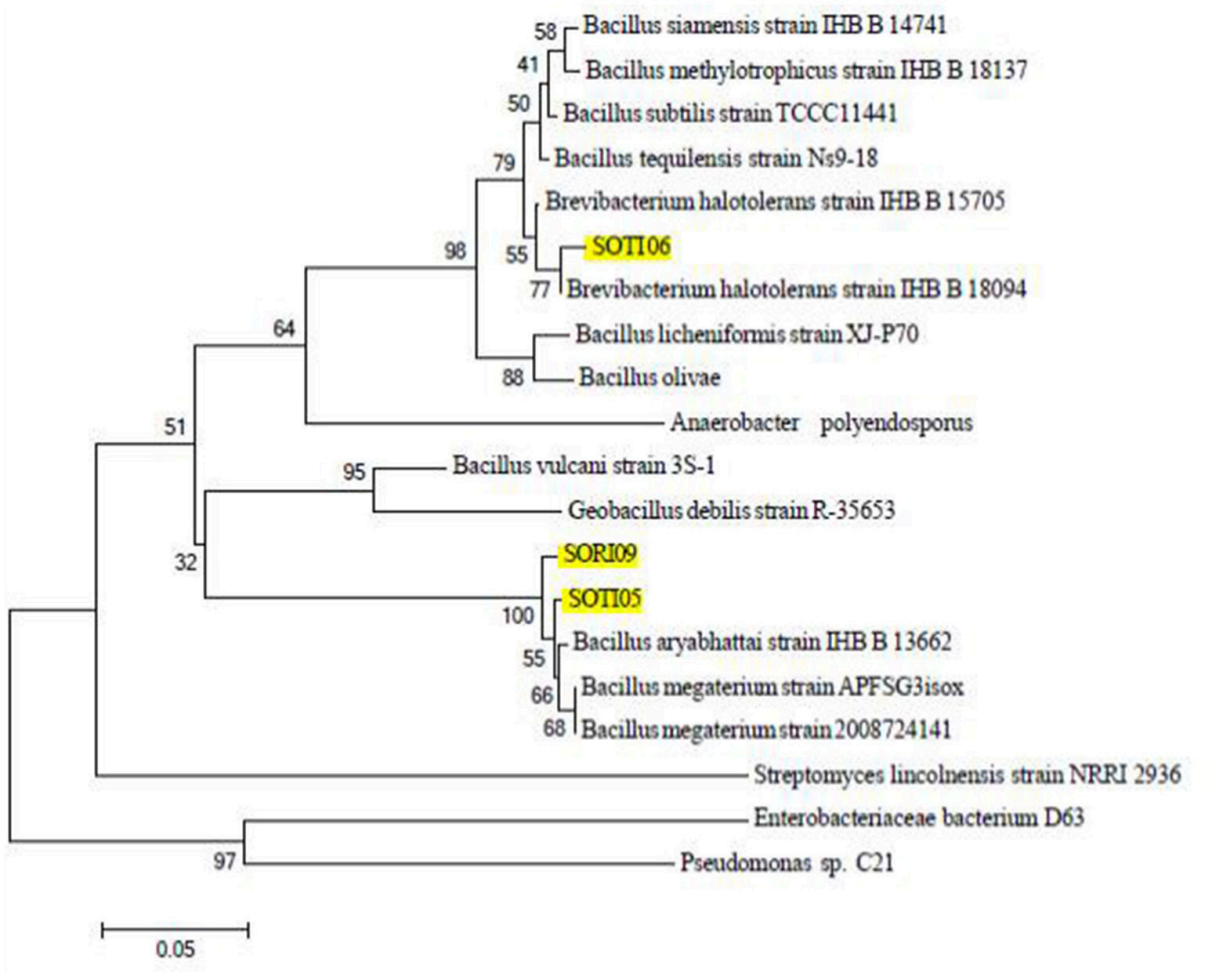
**Phylogeny of CDB isolates**. The phylogenetic relationship of the selected isolates (SORI09, SOTI05, and SOTI06) and their related strains based on 16S rRNA sequence was constructed using Mega 6. Numbers at nodes indicate the level of bootstrap support (1000 replications). Scale bar indicates base substitutions/1000 bases.

Identification of the three promising isolates based on 16S rRNA gene sequence revealed that SOTI06 showed 93% similarity to *Brevibacterium halotolerans* DSM 8802 as their closest organism. SOTI05 showed 100% similarity to *Bacillus megaterium* NBRC 15308 and SORI09 showed 99% similarity to *Bacillus aryabhattai* B8W22, respectively (Figure 4). Genbank accessions for 16S rRNA gene sequence of these isolates, SOTI06, SOTI05, and SORI09 were KX443712, KX443711, and KX443710, respectively.

### Surface attachment of *Brevibacterium* sp. SOTI06

Microbial mediated calcite dissolution starts with surface attachment of the bacteria by means of biofilm formation and EPS production subsequently the mineral dissolution by secreting organic acids, siderophore, and phosphatase. *Brevibacterium* sp. SOTI06 was able to form higher amount of biofilm when supplemented with CaCO_3_ than medium without CaCO_3_ which was evidenced with the increase in OD_600nm_ of former (0.21) than later (0.16). Similarly, the production of EPS was higher (13.3 μg.ml^−1^) in the medium supplemented with CaCO_3_ than in control (4.39 μg.ml^−1^).

### Calcite dissolution

#### Estimation of dissolution

**Figure 5:**
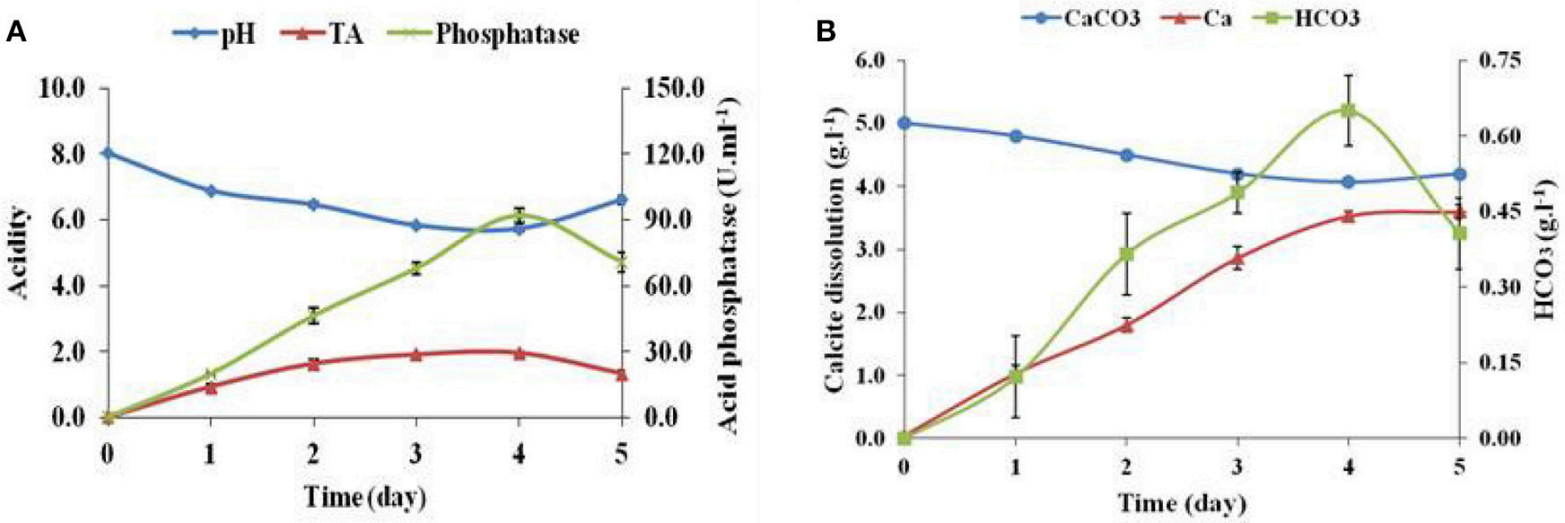
**Calcite dissolution of ***Brevibacterium*** sp. SOTI06**. Acidity in terms of pH and TA (g.l^−1^) indicated their inverse proportion and increase in acid phosphatase activity (U.ml^−1^) **(A)**; Calcite dissolution (CaCO_3_ content and Ca^2+^ release) and HCO_3_ **(B)** of *Brevibacterium* sp. SOTI06 over the period of 5 days. The pH was decreased from day 0 to 4. Whereas, the Ca^2+^ and HCO_3_ content increased over a period and a decrease in CaCO_3_ content indicated that dissolution occurred by the bacterium. Means of three replicate values plotted and error bars indicate the standard error.

The calcite dissolution behavior of *Brevibacterium* sp. SOTI06 was estimated over a period of 5 days by measuring the pH, TA, phosphatase, CaCO_3_, Ca^2+^, ${\text{CO}}_{3}^{2-}$, and ${\text{HCO}}_{3}^{-}$ content in the medium. The results revealed that pH decreased gradually from 8.02 to 5.72 until 4th day and there after increased to 6.60 on 5th day. On contrary, TA and phosphatase activity showed an increasing trend from 0 to 4th day and decreased later. Production of TA started on 1st day (0.93 g.l^−1^) and almost doubled on 2nd day (1.63 g.l^−1^), further an increment in TA was noticed up to 4th day (1.95 g.l^−1^) and suddenly dropped to 1.33 g.l^−1^ at 5th day. But, the phosphatase activity was linearly increased from 1st day (19.7 U.ml^−1^) onwards, reaching maximum up to 4th day (91.8 U.ml^−1^) and reduced to 70.7 U.ml^−1^on 5th day (Figure 5A). The protein concentration was also increased from 1st day (10.0 g.l^−1^) to 4th day (34.4 g.l^−1^) and declined on 5th day (26.7 g.l^−1^).

The supplemented calcium carbonate content was slowly decreased from 5.0 to 4.07 g.l^−1^ with simultaneous increase in calcium and bicarbonate concentrations. The release of Ca^2+^into the solution was higher than bicarbonate ions. A minimal amount of calcium (0.04 g.l^−1^) and no bicarbonates were released on 0th day and thereafter, the release of calcium content was higher until 5th day (3.60 g.l^−1^). However, the bicarbonate content was increased until 4th day reaching the maximum of 0.65 g.l^−1^ and decreased later. Overall, *Brevibacterium* sp. SOTI06 was capable of dissolving 18.6% of calcite within 5 days of incubation (Figure 5B).

#### Organic acid production

**Figure 6:**
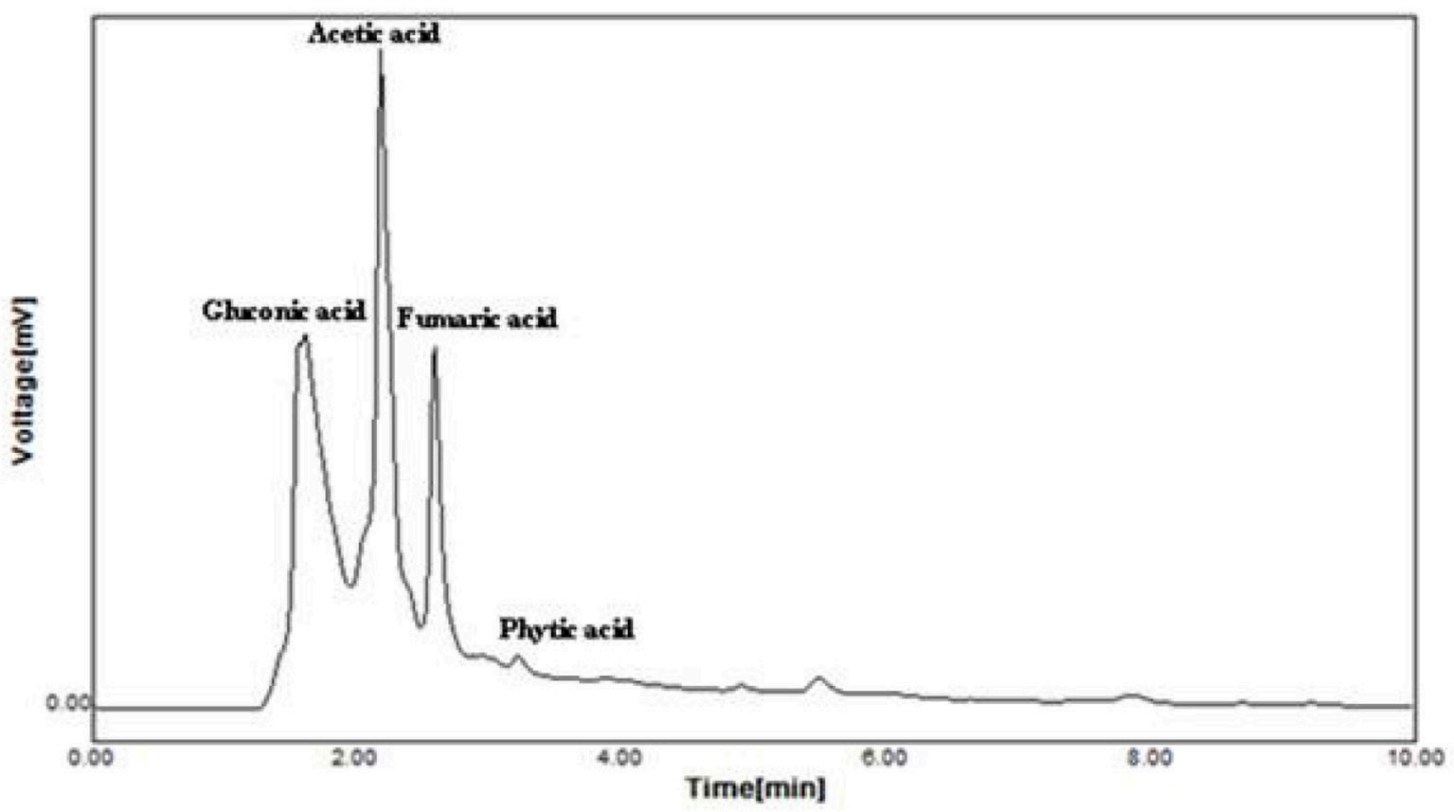
**Organic acid production by ***Brevibacterium*** sp. SOTI06**. The organic acid production was estimated for the culture supernatant using HPLC analysis and the chromatogram showed the acids production. The standards used were gluconic acid, acetic acid, fumaric acid, and phytic acid.

Supplementation of CaCO_3_ to *Brevibacterium* sp. SOTI06 resulted in the production of organic acids such as gluconic acid, acetic acid, fumaric acid, and phytic acid. Among the secreted organic acids, gluconic acid was the predominant one (3.24 mg.ml^−1^) followed by acetic acid (3.17 mg.ml^−1^). A minimal amount of phytic acid (10 μg.ml^−1^) and fumaric acid (7 μg.ml^−1^) was also recorded in the medium enriched with CaCO_3_ (Figure 6). Conversely, the medium without CaCO_3_ resulted in lesser production of acetic acid (0.92 mg.ml^−1^) and fumaric acid (0.25 μg.ml^−1^) whereas; the release of predominant gluconic acid was not observed (data not given).

#### FT-IR analysis

**Figure 7:**
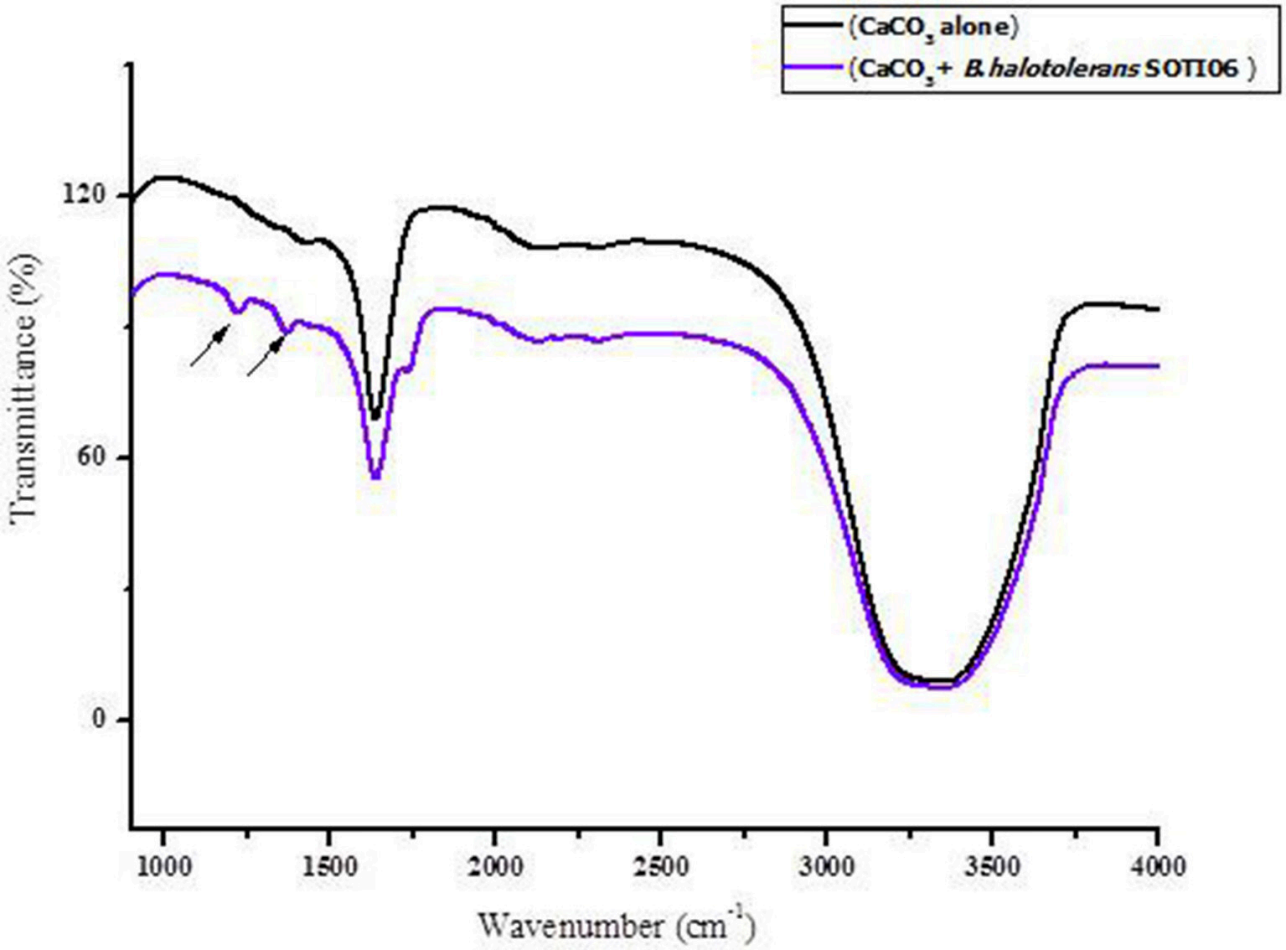
**FT-IR spectrum of calcite dissolution by ***Brevibacterium*** sp. SOTI06**. The spectra in black color indicates control (CaCO_3_ alone) whereas purple color indicates the *Brevibacterium* sp. SOTI06 inoculated sample.

FT-IR spectra of calcite dissolution by *Brevibacterium* sp. SOTI06 was compared with uninoculated control (Figure 7) and the spectral data showed changes in vibration and alteration of structure with reduced intensity (55.36–41.27%) which confirmed the calcite dissolution by bacterium (1636 cm^−1^). Further, the presence of additional two new peaks at wave number of 1222 and 1370 cm^−1^ with strong OH groups was observed in treated sample (Table 1).

**Table 1:** **FTIR spectrum of ***Brevibacterium*** sp. SOTI06**.

**Sample**	**Wavenumber (cm^−1^)**	**Functional group**	**Bond**	**Intensity**	**Mode**
Control	1636	Alkene	C=C	Variable	Stretching (non-conjugated C=C)
Treated	1636	Alkene	C=C	Variable	Stretching (non-conjugated C=C)
	1370	OH	NH	Medium to weak	Amide III combination of C-N stretching and N-H bending

#### Siderophore production

**Figure 8:**
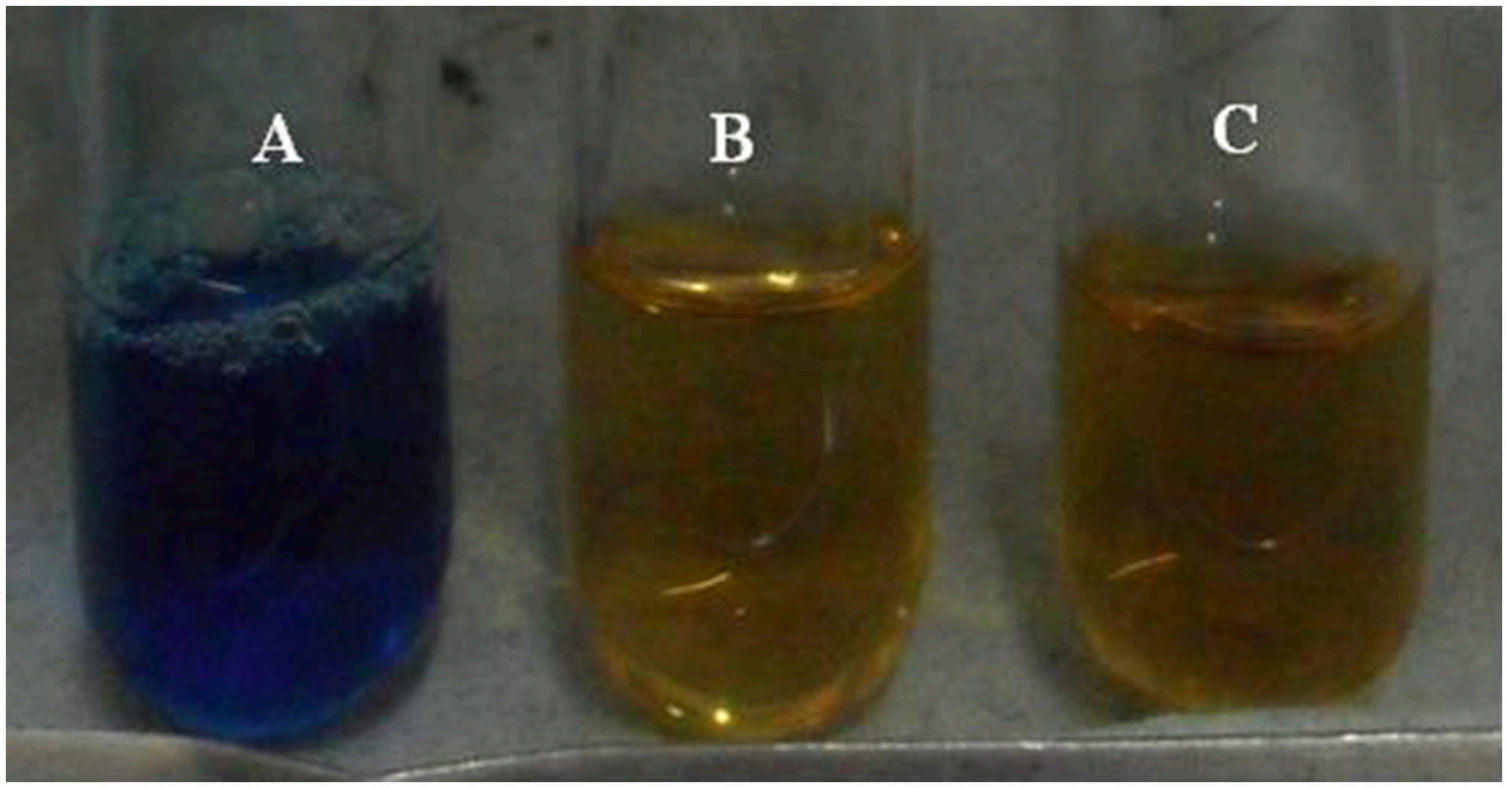
**Siderophore production by ***Brevibacterium*** sp. SOTI06**. The siderophore production was estimated by CAS assay with reference **(A)** in the presence **(B)** and absence **(C)** of CaCO_3_. The change of color from blue to yellow indicated the siderophore production.

The development of yellow halo around the colonies in CAS plate was confirmed by broth assay showed that *Brevibacterium* sp. SOTI06 produced siderophore both in CaCO_3_ amended as well as unamended liquid medium was evidenced by a change of color from blue to yellow (Figure 8). But, their production was higher in amended medium registering 91.6 per cent siderophore units than the control (88.6%).

## Discussion

Salt affected soils are wide spread in many arid and semiarid regions which are the major constraints for agricultural expansion and productivity. The main reason for the increased sodicity is due to faulty irrigation and drainage practices which leads to soil degradation and ultimately reduces crop yield (Sumner, 1993; Sharma and Rao, 1998; Haynes and Hamilton, 1999; Gharaibeh et al., 2011). In order to reduce the sodicity in calcareous soils, the native calcite need to be dissolved to release adequate Ca^2+^ so as to replace the Na^+^ ions, which can be leached out through irrigation (Oster, 1982; Shainberg et al., 1989; Qadir and Oster, 2002). Microbial mediated calcite dissolution studies are very sparse in the literature for instance, calcite and dolomite dissolution was studied in *Shewenella oeindeisis* MR1 (Davis et al., 2007), *Bacillus subtilis*, and *Burkholderia fungorum* (Friis et al., 2003; Jacobson and Wu, 2009). Recently, *Brevibacterium* sp. was isolated from Krast caves and reported their calcite dissolution ability (Sonntag, 2015). Hence, it is imperative to develop calcite dissolving microbes and understanding its mechanisms of dissolution so as to reclaim the calcareous sodic soils effectively. In this contest, the present study on isolation, screening and identification of CDB and understanding the mechanism underpinning calcite dissolution is significant.

The present investigation indicated SOTI06 as the best isolate based on the calcite dissolving ability and titratable acidity. The 16S rRNA gene sequence of the newly isolated bacterial isolate SOTI06 was analyzed to establish its phylogenetic relationship, which showed only 93% similarity with *B. halotolerans* strain DSM 8802 suggesting that this isolate might be a new one and needs further systematic and taxonomical studies to reveal its novelty.

Calcite dissolution is regulated by a wide range of molecules like organic acids, amino acids and these molecules inhibit calcite growth thereby promoting dissolution (Teng et al., 2006). The growth, planktonic form of biofilm formation and EPS production are the mechanisms by which microorganisms attached to the mineral surface (Banfield et al., 1999; Kraemer, 2004; Peacock et al., 2004; Buss et al., 2007; Yi et al., 2008; Shirvani and Nourbakhsh, 2010; Parrello et al., 2016) and helps dissolution. In the present study, the CDB isolate *Brevibacterium* sp. SOTI06 produced considerable amount of EPS and planktonic form of biofilm in the presence of calcite suggesting its possible attachment to dissolute calcite as evidenced by Bissett et al. (2011).

A reduction in pH as induced by the production of TA by *Brevibacterium* sp. SOTI06 determines the solubility of minerals (Whitelaw et al., 1999; Ogbo, 2010; Barroso and Nahas, 2013). The trend of decrease in calcium carbonate content and increase in Ca^2+^ supply from first day onwards indicates that the dissolution process initiated upon inoculation as evidenced from the gluconic acid production by *Brevibacterium* sp. SOTI06 and suggests that the gluconic acid might be the predominant one involved in calcite dissolution. The increase in calcium carbonate content on fifth day might be due to precipitation and these results are in accordance with Subrahmanyam (2013). In multicellular organisms like sponges, the cellular attachment on mineral surface, penetration, and dissolution of calcareous substrates are mediated by many enzymatic activities particularly carbonic anhydrase and acid phosphatases (Kreitzman and Fritz, 1970). In the present CD experiment, the enhanced acid phosphatase activity coupled with drop in pH, CaCO_3_, and release of Ca^2+^ explains the role of this enzyme on supplying calcium through effective calcite dissolution.

The FT-IR results suggest that bacterial dissolution might have altered the structure of calcite and resulted in vibration change. Since the *Brevibacterium* sp. SOTI06 secreted gluconic acid and other acids, which might have facilitated the release of Ca^2+^ from calcite and results in overall mass reduction. Such reduction was evident in the FT-IR spectra of treated samples and also the results with measurement of Ca^2+^ supported this phenomenon. The presence of two additional peaks with strong OH groups might be attributed to acids secreted by *Brevibacterium* sp. SOTI06. From the FT-IR study, the dissolution behavior and acid secretion of *Brevibacterium* sp. SOTI06 was confirmed.

Though, the present study showed calcite dissolution of the isolate under *in vitro* condition, the potential of this bacterium is yet to be evaluated in detail under *in vivo* condition to remediate the calcareous sodic soils.

## Conclusion

The present study reported a calcite dissolving *Brevibacterium* sp. SOTI06 with a potential to dissolute 18% calcite with a simultaneous release of Ca^2+^ ions under *in vitro* conditions. Gluconic acid production, biofilm formation, production of siderophore, and EPS by *Brevibacterium* sp. SOTI06 might be the possible mechanisms attributed to the dissolution of calcite.

## Author contributions

SU and CT conceived the idea and designed experiments. ST conducted the experiments, analyzed the data and helped in drafting the manuscript. SU finalized the results after compiling data and completed the manuscript preparation.

### Conflict of interest statement

The authors declare that the research was conducted in the absence of any commercial or financial relationships that could be construed as a potential conflict of interest. The handling Editor declared a shared affiliation, though no other collaboration, with the authors and states that the process nevertheless met the standards of a fair and objective review.

## Abbreviations

−: Absence
+: Presence
μl: Microliter
16S rRNA: Ribosomal Ribo Nucleic Acid
Amp-X-gal-IPTG, Ampicillin: 5-bromo-4-chloro-3-indolyl-β-D-galactopyranosid and Isopropyl β-D-1-thiogalactopyranoside
Ar: Absorbance of reference
As: Absorbance of sample
FT-IR: Fourier Transformation Infra-Red spectrophotometer
C: Control
Ca^2+^: Calcium ions
CaCO_3_: Calcium carbonate
CAS: Chrome Azurol S
CD: Calcite dissolution
CDB: Calcite dissolving bacteria
cm: Centimeter
${\text{CO}}_{3}^{2-}$: Carbonate
CS: Colony size
CZ: Clear zone
DB: Devenze-Bruni
DNA: Deoxyribo Nucleic Acid
EPS: Exopolysaccharide
g: Gravity
g.l^−1^: Gram per litre
H_2_SO_4_: Sulphuric acid
HCl: Hydrochloric acid
${\text{HCO}}_{3}^{-}$: Bicarbonate
HPLC: High Performance Liquid Chromatography
IAA: Indole acetic acid
M: Molarity
min: Minute
ml: Millilitre
Na^+^: Sodium ion
NaCl: Sodium chloride
NaOH: Sodium hydroxide
nm: Nanometer
OD: Optical density
PCR: Polymerase chain reaction
rpm: Rotation per minute
SI: Solubilization index
SOCE: Sodic soil Coimbatore district Enriched sample
SOCI: Sodic soil Coimbatore district Initial sample
SORE: Sodic soil Ramnad district Enriched sample
SORI: Sodic soil Ramnad district Initial sample
SOTE: Sodic soil Trichy district Enriched sample
SOTI: Sodic soil Trichy district Initial sample
T: Treated
TA: Titratable acidity
UV-VIS: Ultra violet-visible
Zn: Zinc
ZnO: Zinc oxide.
